# miRNAs, Melanoma and Microenvironment: An Intricate Network

**DOI:** 10.3390/ijms18112354

**Published:** 2017-11-07

**Authors:** Gabriele Romano, Lawrence N. Kwong

**Affiliations:** Department of Translational Molecular Pathology, The University of Texas MD Anderson Cancer Center, Houston, TX 77030, USA; LKwong@mdanderson.org

**Keywords:** miRNA, melanoma, tumor microenvironment, epithelial to mesenchymal transition (EMT), immune system, hypoxia, metabolism

## Abstract

miRNAs are central players in cancer biology and they play a pivotal role in mediating the network communication between tumor cells and their microenvironment. In melanoma, miRNAs can impair or facilitate a wide array of processes, and here we will focus on: the epithelial to mesenchymal transition (EMT), the immune milieu, and metabolism. Multiple miRNAs can affect the EMT process, even at a distance, for example through exosome-mediated mechanisms. miRNAs also strongly act on some components of the immune system, regulating the activity of key elements such as antigen presenting cells, and can facilitate an immune evasive/suppressive phenotype. miRNAs are also involved in the regulation of metabolic processes, specifically in response to hypoxic stimuli where they can mediate the metabolic switch from an oxidative to a glycolytic metabolism. Overall, this review discusses and summarizes recent findings on miRNA regulation in the melanoma tumor microenvironment, analyzing their potential diagnostic and therapeutic applications.

## 1. Introduction

The understanding of melanoma biology and histopathology has grown exponentially in the last 20 years, and current therapeutic approaches take into account such developments. For example, the knowledge of the BRAF driving oncogenic lesion has guided the advent of BRAF and MEK inhibitors, effective in more than 50% of the treated melanoma patients. More recently, it has been elucidated that the role of the immune system in melanoma therapy is pivotal, and appropriate immune therapies have been developed, such as anti-CTLA4 and anti-PD1. Even so, most patients (50–60%) treated with these agents do not have a durable response [1,2]. Thus, we anticipate that other microenvironmental and genetic factors play an as-yet therapeutically-unrealized role in melanoma biology. One of the common factors in this web of tumor-stromal interactions are microRNAs (miRNAs). miRNAs are small non coding RNAs that inhibit gene expression mainly through translation inhibition or target degradation. miRNAs have emerged as central players in cancer biology and have been demonstrated to be helpful to determine tumor type, prognosis and response, and are intimately involved in both the tumor cell-intrinsic and the microenvironmental communication of pro- and anti-oncogenic signals. In this review, our goal is to present a structured view of how specific miRNAs mediate tumor-stromal communication in three distinct tumor microenvironmental processes: EMT, immune infiltration, and hypoxia. By organizing the current knowledge in such a way, we hope to provide readers with a clearer top-down view, enabling the identification of both key translational strengths and missing knowledge to guide future miRNA research.

## 2. Epithelial to Mesenchymal Transition (EMT), Exosomes and miRNAs: A Complex Web

The epithelial to mesenchymal transition (EMT) has been proposed as one of the key mechanisms of cancer resistance and invasiveness. Although melanomas are not epithelial tumors, they nevertheless exhibit a spectrum of canonical EMT markers that anti-correlate with melanocyte differentiation markers and Mitogen-activated protein kinase (MAPK) inhibitor sensitivity [3,4], strongly suggesting that this EMT signature defines a biologically relevant—and plastic [5,6,7]—melanoma state. In fact, melanoma cells express E-cadherin (CDH1) (which is fundamental for the contact with keratinocytes) [8], and as a consequence they can encounter an EMT-like process consisting of a decreased expression of CDH1 and increased expression of mesenchymal transcription factors like ZEB1, SNAIL1, or TWIST1 [5,7,9,10]. It is widely demonstrated that the EMT process in melanoma can be driven by oncogenic pathways, including MEK-ERK pathway activation [3,4], and moreover that miRNA-mediated regulation can play a critical regulatory role. Below, we discuss miRNAs in several different EMT-related melanoma contexts: downstream of oncogenic pathways, as potential mediators of disease progression and drug resistance, and as components of various cell–cell communication processes.

### 2.1. miRNA-Mediated EMT Regulation: Biological and Clinical Significance

E2F1 is a well-known cell cycle-regulatory transcription factor downstream of Rb that is one of many transcription factors capable of inducing the EMT process in melanoma. Two miRNAs have been shown to regulate the E2F1-driven EMT switch: miR-224 and miR-452 [11]. miR-224 is found to be up-regulated in a large variety of tumors such as glioma, colorectal cancer, renal carcinoma, and others [12,13,14,15,16]. miR-224 is part of a cluster of miRNAs together with miR-452 (*GABRE* intronic region at chromosome Xq28), which is mostly involved in inflammation-related pathologies and is a validated marker for bladder cancer [17]. miR-224/miR-452 expression is activated by E2F1 through transactivation of the *GABRE* gene. E2F1-induced miR-224/miR-452 expression drives the EMT process through the downregulation of *TXNIP* which is responsible for feedback inhibition of E2F1 itself [11].

Second, it is frequent to observe a correlation between specific miRNA expression, disease progression, and EMT. For example, miR-205-5p progressively decreases during the successive stages of melanomagenesis in mice [18]. The induction of miR-205-5p reduces *RAP1A* expression (an EMT-related protein) and consequentially mitigates cell invasiveness, decreases proliferation, and delays tumor onset [18]. Similarly, miR-542-3p has been described as another key regulator of the EMT process as it is strongly downregulated in melanoma tumor cells and tissues compared to healthy counterparts [19]. The forced re-introduction of miR-542-3p was able to inhibit EMT and metastasis formation in a pre-clinical model of melanoma, putatively through the translation inhibition of *PIM1*, a well-known promoter of tumor growth and spreading [19]. Also, miR-9 is downregulated in metastatic melanomas compared to primary tumors. miR-9 is able to downregulate *SNAIL1* and consequently promote *CDH1* expression, inhibiting melanoma cells’ ability to invade [20]. In this mechanism, a fundamental role is played by NF-κB1 (*NFκB1*) which is a central mediator of the whole process. It is interesting to notice that the same miRNA has an EMT-promoting role in breast cancer, highlighting that the same signal can sort opposite effects in different tumoral contexts [20]. These observations suggests that miRNAs capable of modulating EMT may play a role in melanoma progression, though this awaits further validation.

Third, an interesting association is also seen between miRNA expression profiles and EMT-driven drug resistance. Patient derived xenograft and biopsies from patients with acquired BRAFi resistance showed decrease levels of miR-200c and increased expression of BMI1, ZEB2, ABCG5, and other EMT markers [21]. Mechanistically, miR-200c has been shown to mediate the inactivation of MAPK and PIK3/AKT pathway and the downregulation of mesenchymal markers, such as N-Cadherin and MDR1; BMI1 appears to be a critical mediator of this mechanism [22]. As a result, miR-200c has a double controlling role in cell proliferation arrest and invasion inhibition because of its influence on multiple signaling pathways [21]. Consistently, miR200c has been proven to be helpful in inhibiting EMT in other contexts, such as in experimental vaccination against melanoma [23,24]. Moreover, miR200c is also well-established as a central EMT regulator in various other cancers [25,26,27,28].

Fourth, interaction of miRs with epigenetic programming is suggested by the finding that the miR-211 promoter can be methylated by DNA (cytosine-5)-methyltransferase 1 (DNMT1) [29]. EMT and epigenetic reprogramming are also closely linked, and some miRNAs are involved in such epigenetic regulation [30]. For example, the miR-31 locus is frequently lost in melanoma samples and this is correlated with invasion and metastasis of melanoma, in particular because of its regulatory relationship with Enhancer of zeste homolog 2 (*EZH2*) [31]. EZH2 is part of the Polycomb complex 2, which is able to mediate trimethylation of histone 3 at lysine K27 at the miR-31 promoter region [32,33]. Among the genes regulated by miR-31 are *SRC*, *NIK*, *RAB27A* and *MET*, whose products in turn, control transcription factors ultimately regulating EZH2 expression, which is known to be a key regulator of EMT in melanoma [34]. When miR-31 is lost because of a frequent deletion in chromosome 9p, this feed forward loop is de-regulated and an invasive phenotype is promoted [31], positing miR-31 as one of the multiple miRNAs that indirectly regulate EMT. Interestingly, *EZH2* and *MITF* are often reported to be regulated together by miRNAs and linked to de-differentiation, invasion and metastasis. *EZH2* and *MITF*, in fact, are targets of miR-101 and miR-137 that have been associated with the control of the invasive phenotype of melanoma cell lines. Consistently, a low expression of miR-137 and miR-101 is correlated with poor survival in stage IV melanoma patients [35,36].

### 2.2. Long- and Short-Range Cell-Stroma Communication: Exosomes, Integrins and Keratinocytes

An interesting axis of communication has been identified between melanoma cells and keratinocytes of the epidermal layer: when tumor cells come in contact with keratinocytes, Notch pathway activation impairs Microphthalmia-associated transcription factor (MITF) binding and repression of the miR-222/221 promoter, and consequently promotes tumor invasion putatively through *GRB10* or *ESR1* inhibition [37]. MITF is a transcription factor that, when active, keeps melanocytes in a differentiated status. On the contrary, when *MITF* expression is lost or de-regulated during melanoma progression, tumor cells tend to evolve towards a dedifferentiated mesenchymal like phenotype [5,7]. In addition, miR222/221 also promotes EMT in breast cancer through a direct regulation of *ZEB2* [38]. It is interesting to notice that only when melanoma cells activate Notch signaling the MITF/miR-222/221 axis is deregulated and causes tumor invasion, further underlining how fundamental context and microenvironment are in regulation of tumor phenotype [37]. It has also recently been suggested that a crucial part of the EMT process in melanoma may be mediated by exosomes. Exosomes are vesicles generally of the dimension of 50–150 nanometers and are part of the autocrine and paracrine communication among tumor cells and tumor microenvironment [39]. Additionally, it has been shown not only that the EMT process can be mediated by exosomes in a paracrine fashion, but also that a particular miRNA, let7i, is involved in the process [40]. let7i is part of a finely regulated axis with *LIN28B* and *HMGA2*, its two principal targets; it has been speculated that the circuit comprehending *LIN28B* and *HMGA2* can control the EMT process, even if the molecular details of the mechanism are not clear [41,42,43]. MAPK pathway activation was demonstrated to be necessary for the whole process as exosome-mediated EMT was inhibited by MAPKi administration [40]. Surprisingly, exosomes from melanoma tumor cells are also able to model and re-program stromal cells, like fibroblasts, in order to form a promoting niche for tumor invasion. Exosomes are able to influence fibroblasts at distance, and the core actors of reprogramming are miRNAs. miR-211 contained in exosomes, in fact, caused an increase in collagen contraction and expression of the pro-inflammatory genes *IL1, IL6, IL8, CXCL1* and *CXCL2*, transforming resident fibroblasts in cancer-associated fibroblast (CAF), which favor the growth and invasion of melanoma cells [44]. The whole process involves MAPK signaling once again, and in particular *IGFR2*, which is a direct target of miR-211 [44]. Interestingly, miR-211 had already been shown to modulate EMT in melanoma via *RAB22A* expression inhibition [29]. However, the exact role of exosomes in mediating human melanoma progression remains controversial and these observations await further in vivo validation.

Finally, integrins are another category of molecules strongly involved in the EMT process—because of their role in cell adhesion [45]—whose expression is strongly context dependent and directly intertwined with miRNAs. miR-214 expression, for example, is generally low or undetectable in cell culture while it increases dramatically when tumor cells are injected in vivo where it targets *TFAP2C* and *ITGA3* [46]. Consistently, miR-214 was demonstrated to favor the extravasation and metastasis process of melanoma tumor cells, also because it is apparently involved in ALCAM and MET upregulation [47,48]. Similarly, miR125b loss of expression is associated with melanoma progression and invasion. *ITGA9* is the main target of miR-125b, which mediates a consistent regulation of the classic EMT markers such as cadherins, VIMENTIN, and SNAIL [49]. The integrin B3, encoded by *ITGB3*, is also widely known to be overexpressed in melanoma and other solid tumors, and its expression is inhibited by let7a binding to the 3′ untranslated region (3′ UTR) of *ITGB3* [50,51]. When let7a expression is lost or decreased, *ITGB3* and *NRAS* expression boosts and promotes the melanoma invasive phenotype [51]. Interestingly, Integrin-α V (*ITGAV*) is suppressed by miR-146a, a miRNA known to be involved in melanoma cell growth regulation and whose expression is upregulated during melanoma progression. [52,53,54]. As a result, miR-146a is able to exert a paradoxical role in melanoma tumor cells: while it inhibits tumor metastasis through ITGAV inhibition, at the same time it favors tumor growth through the activation of the AKT/PTEN pathway [54].

**Figure 1:**
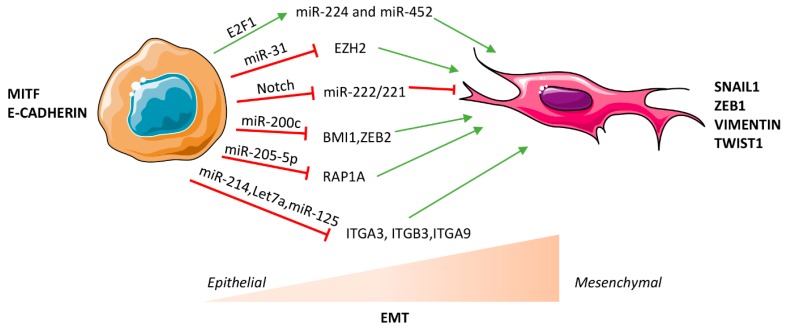
MicroRNA (miRNA)-mediated regulation of epithelial to mesenchymal transition (EMT) in melanoma. miRNAs can facilitate or impair EMT through transcriptional, post-transcriptional or epigenetic mechanisms in a context dependent manner. Red T symbols indicate repression, green arrows indicate up-regulation.

Overall, miRNAs act at different levels of intra- and extra-cellular communication among the multiple cell types present in the melanoma tumor niche, mainly as indirect regulators of EMT transcription factors. Additionally, miRNAs appear to be interrelated with the MAPK pathway that is predominant in melanoma and capable of inducing EMT (Figure 1). However, a major effort remains to tease out exactly which miRNAs act proximally, distally, or even within the EMT induction program itself. Such a knowledgebase will allow the field to predict epistatic interactions and to guide the identification of feasible miRNA-based biomarkers and putative therapeutic interventions.

## 3. miRNAs and Regulation of the Immune Dynamics

The role of the immune system in melanoma is widely known and studied. Many effective therapeutic approaches rely on the knowledge of the immune dynamics in melanoma growth and development. Indeed, an efficacious immune response against the tumor is intimately associated with achieving a durable and long lasting effect [1,2]. Recently, some evidence has been accumulated about the immune suppressive/evasive effect of families of miRNAs.

### 3.1. miRNA and Adaptive Immunity: Regulation of T Cell Activity

miR-30b/miR-30d is involved in the melanoma metastatic process, but not in the classic EMT invasive pathways, and instead have the immune-stimulatory GalNAc transferases (GalNAc-Ts) *GALNT7* and *GALNT1* as targets, among others. Specifically, miR-30d-mediated *GALNT7* inhibition stimulates the expression of the immune suppressive IL-10 cytokine which in turn triggers an immune suppressive microenvironment as measured by an increase in the number of FoxP3^+^ cells [55]. The creation of an immune-privileged microenvironment favors the escape of tumor cells from immune surveillance and facilitates invasion [56]. The fine biochemical balance of tumor milieu is, in fact, a determining factor for melanoma development and progression. Hypoxia, for instance, can strongly influence tumor growth, differentiation and development ([57,58] and next section). miR-210 is among the hypoxia-induced miRNAs in melanoma and is able to impair susceptibility to T-cell lysis by tumor cells [59]. Interestingly, miR-210 does not target major hiscompatability class I (MHC class I) molecule genes or any other gene associated with cell recognition, and it has no effect on hypoxia transcription factors such as *HIF1α* or *HIF2α*. Contrarily, miR-210 inhibition of *PTPN1, HOXA1,* and *TP53I11* has been showed to have a great influence on Cytoxic T Lymphocytes (CTL) lysis: miR-210 knock down restores the sensitivity to CTL lysis by tumor cells, likely through the stimulation of TNF-α, IL-6, and IFN-β.

Another player in the miR-related melanoma immune regulation process is ADAR1, which is a member of the family of adenosine deaminases that act on RNA (ADARs) [60,61]. Suppression of *ADAR1* expression causes tumorigenesis and metastasis in melanoma through a mechanism that involves miR-455-5p and CREB [62]. Recently, ADAR1 has been suggested to be important also in the mechanism of recognition of tumor cells by T cells. This last phenomenon is cell-contact and, more specifically, ICAM1 dependent. ADAR1 controls the transcriptional levels of miR-222 that, in turn, inhibits *ICAM1* expression. Consistently, miR-222 expression was detected to be inversely related to clinical efficacy of ipilimumab in melanoma patients [63].

In the context of High-intensity focused ultrasound (HIFU) therapy in melanoma preclinical models, it was found that among HIFU-induced-benefits there is a stimulation of immune response to the tumor that is miR-dependent. HIFU-stimulated IFN-gamma and TNFα induction and increased CD86 expression in tumor tissue; this was mediated by miR-134 whose direct targets include CD86 [64]. CD86 is an important co-stimulatory molecule and, when it is lacking, T lymphocyte activation is rendered more difficult and less probable [65].

### 3.2. miRNAs and Immune Suppression/Evasion: Focus on Innate Immunity

In addition to adaptive immunity, the innate components of the immune system, and in particular myeloid lineage cells, play a relevant role in regulation of melanoma dynamics [66]. More specifically, miRNAs were found to be associated in the regulation of natural killer (NK) cells, macrophage and myeloid-derived suppressor cell (MDSC) immune responses. For example, CSF1-ETS2 pathway activation induces miR-21, miR-29a, miR-1423p and miR-223 in macrophages [67]. CSF1-ETS2 axis mediates tumor-promoting M2 reprogramming of macrophages and it was demonstrated that miR-29a and miR21 target anti-angiogenic modulators and genes involved in M1 polarization (*PDCD4, SPRY1, TIMP3* by miR-21 and miR-29a targets *COL4A2, SPARC* and *TIMP3)* influencing melanoma tumors growth and metastasis. Consistently, miR-21 and miR-29a are highly expressed in specific suppressive myeloid populations in mouse bone marrow and patient blood during melanoma metastatic progression [67].

NKG2D ligands (NKG2DL) of the NKG2D receptor are generally a sensitizing factors to tumor cell killing by NK cells. Nonetheless, there is a process called “shedding” that increases the soluble levels of NKG2DL, impairing the effective killing of tumor cells by NK cells [68,69]. ULBP2 is a NKG2DL whose high expression in sera of patients is associated with poor prognosis. miR-34a/c and miR-449a/miR-449c bind to 3′ UTR of *ULBP2*, downregulating its expression and are hypothesized to be involved in the process of melanoma cell recognition by NK cells [70].

Another well-studied miRNA is miR-155. Its role in immune regulation is controversial, as it has been associated with the promotion of immune activation, but it has also been recently conjectured to have an immune suppressive role. miR-155 is processed from the B cell integration cluster, a noncoding transcript primarily upregulated in both activated B and T cells, and in monocytes/macrophages upon inflammatory stimuli [71,72]. miR-155 increases T cell immune reactivity against tumors in lymphoreplete hosts [73] and it has been reported to aid immunity against tumors in different contexts [74,75]. Even if miR-155 has been found associated with immune stimulatory pathways, it is also able to exert immune suppressive functions depending on the context. In melanoma models, miR-155-induced MDSC recruitment may be required for their suppressive function [76]. Mechanistically, miR-155 upregulation in MDSCs appears to induce immune suppressive phenotypes through the inhibition of *SOCS1*, a negative regulator of the JAK-STAT pathway [76]. What is notable is that miR-155 upregulation is favored by IL1β, and this mechanism can be a way to circumvent immune recognition [77]. MSDC functions and biological mechanisms are far from being completely elucidated, but it is becoming more and more evident that their role in the abrogation of immune response is pivotal [78,79] and miRNAs seem to have a part in mediating their effects. For example, MSDCs immune suppressive nature is exploited by miR-494. miR-494 induces CXCR4-mediated chemotaxis and is able to influence survival of MDSCs through PTEN inhibition. Interestingly, miR494 expression in MDSCs is induced by melanoma tumor cells through TGFβ1 secretion [80].

Experimental models for ultra violet radiation (UVR)-induced melanoma have also highlighted that UVR-induced inflammation can promote immune-evasion. Exposure to UVR is, in fact, a broadly studied core phenomenon in melanoma development. It is widely known that UVR has a direct mutagenic role in disease, as evidenced by the discovery of an elevated number of transitions throughout sun-exposed melanoma genomes [81]. An interesting network existing between UV-inhibited miRNAs and immune evasive genes has been depicted: a complex web of 14 miRNAs has been hypothesized to be altered after UV exposure, leading to the increase of immune evasive molecules such as CCL2, CCL8, PD1 and B7H2 [82].

Recently, it has been suggested that miRNAs can also be involved in immune checkpoint regulation [83]. miR-28 expression, for example, has been found reduced in 30% of exhausted T-cells in melanoma. miR-28 binds the 3′ UTR of *TIM3*, *BTLA* and *PD-1*. If mir-28 mimetics are administered to exhausted T-cells the phenotype can be reverted, restoring IL-2 and TNF-α production [84]. miR-17-5p has also been associated with the regulation of checkpoint inhibitor molecule PD-L1: BRAF inhibitor resistant melanomas bear increased expression of PD-L1; such increase is inversely correlated with patient plasmatic levels of mir-17-5p which has PD-L1 as a direct post-transcriptional target [85].

**Figure 2:**
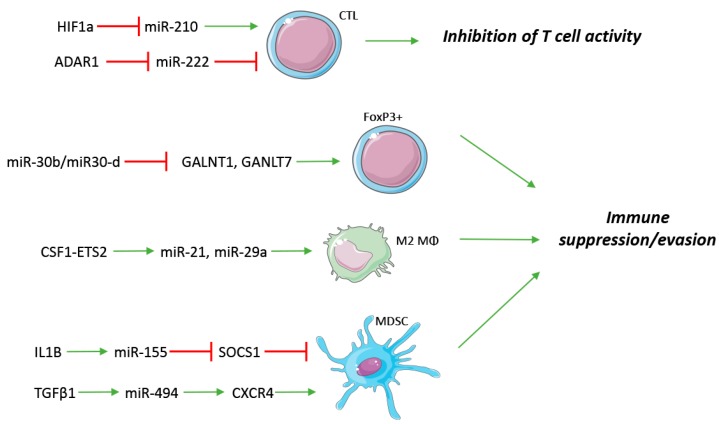
Innate and adaptive immune regulation by miRNAs. miRNAs are involved both in soluble factor and receptor regulation of immune cells, frequently exerting an immune suppressive/evading phenotype. CTL: Cytotoxic T Lymphocyte; FoxP3^+^: Regulatory T cells; M2 Mϕ: M2 Macrophage; MDSC: myeloid-derived suppressor cell. Red T symbols indicate repression, green arrows indicate up-regulation.

In conclusion, miRNAs are emerging as relevant actors in immune regulation and, more specifically, they often appear to mediate the exploitation of a suppressive/evasive phenotype. They do so by participating in the homeostatic processes of the immune system, at various levels; when a perturbation of the microenvironment intervenes (hypoxia, UVR, etc.), or miRNA-expression is compromised, the fine regulation of the physiologic processes can be lost and can give rise to an immune-compromised tumor niche (Figure 2). Now that specific miRs have been implicated in various adaptive and innate immune settings, a comprehensive understanding of their coordination is needed to deconvolute likely biomarkers and therapeutic intervention points.

## 4. miRNAs, Hypoxia and Melanoma Metabolism

Tumor cells’ ability to survive in their microenvironment is dictated by their ability to adapt to various circumstances and change their survival capacities. One of the most typical needs is to adapt to hypoxia, a very frequent phenomenon in tumor settings [57,58].

The two most studied hypoxia-induced transcription factors are HIF1α and HIF2α for whom miR-210 is a direct transcriptional target [86]. miR-210 plays a regulatory role in inhibiting cell cycle arrest in hypoxic conditions, inhibiting MNT, a known MYC antagonist, and favoring the cell growth of tumor cells even in absence of oxygen [87]. As described above, miR-210 also impairs CTL lysis of melanoma cells, decreasing tumor cells’ sensitivity to this process. This is in turn is triggered by hypoxia linking the immune escape mechanism to oxygen deprivation [59]. Even in normoxic environment, miR-210 has been found to be upregulated together with miR-224, miR-452, and miR-218 in a HIF1α-dependent manner, causing an increase in *BNIP3* and *ATF3*, response genes that react to oxygen deprivation [88]. Consistently, miR-210 was found to be increased in the plasma of metastatic melanoma patients [89].

Contrariwise, miR-33a/b [90] and miR-18b have been reported to have HIF1α as direct target; their expression causes cell growth inhibition and is generally correlated with a better prognosis. miR-18b expression, in particular, causes cell cycle arrest through glycolysis inhibition [91].

An additional actor in the hypoxia mechanism is miR-211, known to be involved in melanoma cell proliferation and invasion [92]. miR211 is able to sensitize melanoma cells to hypoxic conditions, inhibiting HIF1α induction under oxygen deprivation. miR-211 acts as a metabolic switch increasing oxygen consumption and downregulating *PDK4* expression. Melanoma cells often bear a very low expression of miR-211, which leads to increased *PDK4* expression and consequent decreased Pyruvate dehydrogenase (PDH) activity, which in turn downregulates the tricarboxylic acid (TCA) cycle and oxidative phosphorylation by mitochondria. The whole phenomenon favors tumor cell survival in low O_2_ hostile environments [92].

It important to notice also that in the presence of O_2_, tumor cells often switch towards a glycolytic metabolism (aerobic glycolysis) [93,94]. This phenomenon is known as the “Warburg effect” and one of the advantages for the tumor cell is likely to be the faster accumulation of biomass despite the inefficiency of the metabolic process. miRNAs can move the balance towards a mainly glycolytic metabolism, inhibiting oxidative phosphorylation (OxPhos), or vice versa. For example, in melanoma cell lines, let7a inhibits some key anabolic enzymes such as G6PD, inosine monophosphate dehydrogenase (IMPDH2), Fatty Acid Synthase (FASN), stearoyl-CoA desaturase (SCD), and 4-phosphopantetheinyl transferase (AASDHPPT), leading to OxPhos and consequent oxidative stress induction [95].

Autophagy is an additional mechanisms of survival that can be enacted by both healthy and tumor cells during stressful conditions such as starvation or hypoxia [96,97]. This complex cellular process involves key proteins such as BECLIN-1, ATG5 and UV Radiation Resistance Associated (UVRAG) which are direct targets of miR-216b [98]. Autophagy upregulation in tumor cells can lead to increased survival of cancerous cells and ultimately drug resistance [99]. BRAF inhibition in melanoma, for example, has been shown to downregulate mir-216b expression, promoting autophagy. Remarkably, the co-administration of miR-216 with a BRAF inhibitor was able to increase drug efficacy in vivo, inhibiting autophagy mediated drug resistance [98]. A restricted supply of oxygen and nutrients can have multiple effects, not only on cancer cells, but also on stromal and immune components of the microenvironment. An interesting phenomenon, for example, involves the metabolic T cells linked to the EZH2 transcription factor. We previously discussed the relevance of EZH2 in the EMT process, but this transcription factor exerts a pleiotropic effect also on T cell phenotypes. More specifically, EZH2 suppresses Notch repressors (*NUMB* and *FBXW7*) via trimethylation of histone H3 at Lys27, stimulates T cell polyfunctional cytokine expression, and promotes their survival via Bcl-2 signaling. Intriguingly, in the context of glucose restriction, tumors are able to impair T cell functionality in ovarian cancer and melanoma models trough miR101 and miR26a (EZH2 repressors) [100].

Another typical way to react to the absence of oxygen and nutrients in the tumor is the promotion of neo-angiogenesis [101,102]. miR-1908, miR-199a-5p, and miR-199a-3p have been identified as key regulator of the process in melanoma. These miRNAs target apolipoprotein E (ApoE) and the heat shock factor *DNAJA4*, which promotes ApoE production. ApoE suppresses invasion and endothelial recruitment specifically by engaging melanoma cell LRP1 and endothelial cell LRP8 receptors, respectively [103]. It has been shown that miR-1908, miR-199a-5p, and miR-199a-3p are robust prognostic and therapeutic targets in melanoma, in reason of this finely regulated mechanism [103].

**Figure 3:**
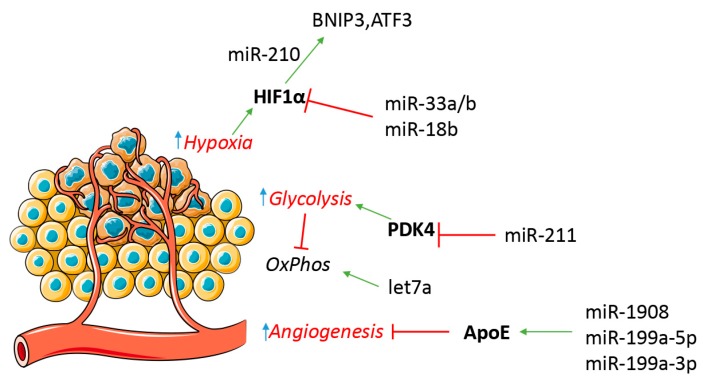
miRNA participate in melanoma metabolic regulation. Hypoxia can favor a tumor metabolic switch from an oxidative metabolism (OxPhos) to a glycolytic one. miRNA are involved in tumor cell response to hypoxia and neo-angiogenesis. Red T symbols indicate repression, green arrows indicate up-regulation.

Comprehensively, various families of miRNAs are involved in the metabolic regulation of tumor cells under stressing conditions. Hypoxia or nutrient deprivation can be crucial triggers for tumor cell metabolic change, which implies a profound modification and evolution of tumor cells and a challenge to their ability to adapt (Figure 3). There is some evidence showing that hypoxia can even impact global miRNA expression [86,104,105], but additional research is needed to further substantiate this claim.

It is interesting to notice that miRNAs can often tip the balance towards a glycolytic or an oxidative metabolism, effectively determining the fate of tumor cells survival and/or the ability of the immune system to effectively eradicate the lesion.

## 5. Conclusions

Much of the miRNAs biology is still obscure, but there is increasing evidence that they participate in many crucial cancer phenomena. Our structured analysis of miRNAs mediating the tumor-stromal interaction in melanoma suggests that miRNA actions both closely within the tumor microenvironment and at a distance can provide fine-tuning of cancer phenotypes. For example, exosomes are capable of transporting miRNAs at great distances, eliciting EMT-related or other signaling to create tumor-friendly niches. Close-range interactions can be exemplified with, for example, miRNA-mediated modification of M2 macrophages or MDSCs to regulate T cell behavior and immune checkpoints and thus the immune-suppressive/evasive status of the tumor. Another close-range interaction is typified by miRNAs that often mediate the metabolic switch in melanoma, regulating the expression of some of the key enzymes of the glycolytic or oxidative phosphorylation chains to create cancer-friendly metabolic states. This bird’s eye view of melanoma microenvironmental miRNA interactions contextualizes the various translational strengths of miRNAs, to wit: miRNAs can be (1) sampled in relevant microenvironmental milieu such as blood and lymph and purified from exosomes and similar structures; (2) easily incorporated into gene expression signatures through pan-RNA platforms such as RNA deep sequencing (RNAseq); (3) detected as cell-free circulating RNAs; and 4) assayed starting from low amounts of total RNA because of their increased stability compared to mRNAs in both plasma and formalin-fixed samples [106].

In order to maximize the translational value of miRNAs, however, many challenges remain to be overcome, both technical and biological. For example, miRNAs have multiple targets and their activity strongly relies on the genetic and microenvironmental background of the tissue/tumor. Moreover, there is increasing evidence that miRNAs can act in non-canonical ways which require further characterization [107]: (1) they can be transcribed from exonic regions [108], (2) they can act directly in the nucleus as pre-miRNA or even be imported back into the nucleus as mature miRNAs from the cytoplasm [109,110,111], and (3) they can bind mRNAs not only at the 3′ UTR but also at the 5′ UTR, or inside the coding sequence, to exert various effects on transcription [112,113]. In addition, still very little is known about possible mechanisms of resistance to miRNA inhibition. Nevertheless, interference with specific miRNAs have shown some potential therapeutic promise in a few clinical settings, [114,115], while their status as clinical biomarkers continues to be increasingly validated [116,117,118,119].

In conclusion, our review lays out a cadre of specific miRNAs with potential functional and prognostic values in melanoma through their interactions with the tumor microenvironment. Such miRNAs have previously been understudied in comparison to those with tumor cell-intrinsic action, and thus, shining a spotlight on such miRNAs with potential clinical relevance is hoped to aid the field in sorting out potential miRNA targets and biomarkers. Particularly with the rise of both targeted and immune checkpoint therapies to the front stage in melanoma, the roles of EMT, regulatory immune cells, and metabolic signatures in drug sensitivity and resistance [120,121,122], and the driving miRNAs behind them have become all the more clinically relevant. We envision miRNAs as a complementary field to therapies that target mRNAs, DNA, and proteins, with the potential for helping to hijack the ability of miRNAs to fine-tune tumor cell survival.
